# Pathological Role of Unsaturated Aldehyde Acrolein in Diabetic Retinopathy

**DOI:** 10.3389/fimmu.2020.589531

**Published:** 2020-10-22

**Authors:** Miyuki Murata, Kousuke Noda, Susumu Ishida

**Affiliations:** ^1^Laboratory of Ocular Cell Biology & Visual Science, Hokkaido University, Sapporo, Japan; ^2^Department of Ophthalmology, Faculty of Medicine and Graduate School of Medicine, Hokkaido University, Sapporo, Japan

**Keywords:** inflammation, oxidative stress, vascular adhesion protein-1, acrolein, diabetic retinopathy, spermine oxidase

## Abstract

With increasing prevalence of diabetes and a progressively aging society, diabetic retinopathy is emerging as one of the global leading causes of blindness. Recent studies have shown that vascular endothelial growth factor (VEGF) plays a central role in the pathogenesis of diabetic retinopathy and anti-VEGF agents have become the first-line therapy for the vision-threatening disease. However, recent studies have also demonstrated that diabetic retinopathy is a multifactorial disease and that VEGF-independent mechanism(s) also underlie much of the pathological changes in diabetic retinopathy. Acrolein is a highly reactive unsaturated aldehyde and is implicated in protein dysfunction. As acrolein is common in air pollutants, previous studies have focused on it as an exogenous causative factor, for instance, in the development of respiratory diseases. However, it has been discovered that acrolein is also endogenously produced and induces cell toxicity and oxidative stress in the body. In addition, accumulating evidence suggests that acrolein and/or acrolein-conjugated proteins are associated with the molecular mechanisms in diabetic retinopathy. This review summarizes the pathological roles and mechanisms of endogenous acrolein production in the pathogenesis of diabetic retinopathy.

## Introduction

The prevalence of diabetes is increasing worldwide (1) and the global prevalence has been estimated at 10.2% (578 million) by 2030 and 10.9% (700 million) by 2045 (2). Diabetic retinopathy (DR) is a retinal microvascular complication caused by diabetes with complex multifactorial pathogenesis. Epidemiological studies have revealed that the prevalence and severity of DR increases with age and duration of diabetes (3). For instance, 23% of patients with type II diabetes have non-proliferative retinopathy after 11–13 years, 41% after 14–16 years, and 60% after 16 years (3). Hence, with increasing diabetes prevalence and a progressively aging society, DR is emerging as one of the global leading causes of blindness.

Early events in DR, such as thickening of the capillary basement membrane and pericyte loss, both of which contribute to vascular instability in the diabetic retina, are well known. The changes in retinal microvasculature are followed by capillary non-perfusion and ischemia-mediated pathology such as pathologic neovascularization originating from retinal vessels. The pathologic neovascularization due to retinal ischemia causes the formation of fibrovascular tissues at the vitreoretinal surface, which is a hallmark of proliferative diabetic retinopathy (PDR), and leads to severe complications such as vitreous hemorrhage and tractional retinal detachment. Furthermore, retinal ischemia compromises the blood-retinal barrier (BRB) and results in fluid accumulation in the center of the diabetic retina, i.e., diabetic macular edema (DME). During the past few decades extensive efforts have been undertaken to develop therapeutic strategies to control vascular complications in DR.

Recent advances in basic research have demonstrated that vascular endothelial cell growth factor (VEGF) plays a major role in the pathogenesis of DR (4, 5) and clinical application of anti-VEGF agents have dramatically improved the therapeutic outcomes of the vision-threatening disease. Nowadays, anti-VEGF agents have become the standard first-line treatment for DR, in contrast to the past where photocoagulation and vitreous surgery for advanced DR were the only options.

However, accumulating evidence from clinical research has highlighted a patient population with DME that is refractory to anti-VEGF therapy (6). In fact, DME patients who suffer from poor visual acuity caused by sustained exudative changes in the macula despite frequent injections of anti-VEGF agents are often encountered in the clinical setting. This indicates that DR is a multifactorial disease and VEGF-independent mechanisms also underlie many of the pathological changes.

Some of the reported pathological signs in DR include chronic inflammation and oxidative stress (7). Macroscopic signs of inflammation such as redness (Rubor), heat (Calor), swelling (Tumor) and pain (Dolor), are not pathological features in the diabetic retina and the classical definition of inflammation is inadequate to describe the characteristics of DR. However, at a microscopic level, inflammatory responses including vessel dilatation, hemodynamic alteration, exudation and leukocyte accumulation/migration are present in retinal and choroidal tissues during development of DR (8). In addition, there is growing scientific evidence that oxidative stress plays a crucial role in the development of diabetic complications including DR (9).

Therefore, elucidation of “VEGF-independent” pathophysiology in DR is critical to fulfill an unmet medical need for patients with DR refractory to anti-VEGF therapy. In this review, we provide our perspective on the VEGF-independent mechanisms in the pathogenesis of DR, with focus on the unsaturated aldehyde acrolein.

## What Is Acrolein?

**Figure 1:**
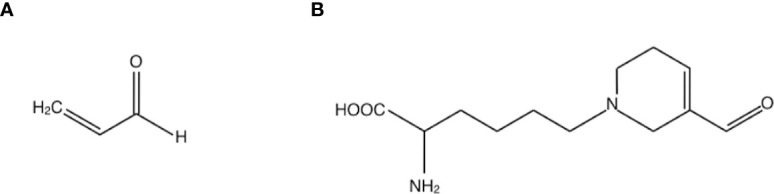
Structures of **(A)** acrolein and **(B)** one of the major acrolein-conjugated proteins, N^ϵ^-(3-formyl-3, 4-dehydropiperidino) lysine adduct (FDP-Lys).

Acrolein is a highly reactive unsaturated aldehyde that causes protein dysfunction by reacting preferentially with Cys, Lys and His residues of peptide chains *via* Michael-type reaction (10, 11) (**Figure 1A**). In acrolein-conjugated proteins, the Lys adduct is a more stable product than the other adducts. One of the major acrolein-Lys adducts, N^ϵ^-(3-formyl-3, 4-dehydropiperidino) lysine adduct (FDP-Lys) (**Figure 1B**), is known as a reactive intermediate that can covalently bind to thiols, including glutathione (GSH), through the retained electrophilic carbonyl moiety (10). Since the reaction of GSH with acrolein and/or FDP-Lys depletes intracellular reserves of GSH, which is a major antioxidant enzyme, acrolein increases oxidative stress by limiting oxidative stress resistance in the body (12). Notably, previous studies have implicated acrolein in a wide range of vascular diseases such as brain infarction (13) and neurodegenerative diseases such as Parkinson’s disease (14) and Alzheimer’s disease (15). Since DR is a representative retinal disease with characteristics of both vascular disease and neurodegenerative disease, in recent years much attention has been paid to acrolein as a possible participant in the pathogenesis of DR.

## Endogenous Acrolein Generation

Since acrolein is common in air pollutants such as cigarette smoke, vehicle exhaust and overheated cooking oil, acrolein was previously focused on as an exogenous causative substance in the research of respiratory diseases such as lung cancer (16). However, recent studies including ours have revealed that acrolein is also generated endogenously through peroxidation of unsaturated fatty acids (17) and polyamine metabolism (18), resulting in cell toxicity and oxidative stress. Among the molecules related to endogenous acrolein generation, the current review focuses on two major enzymes, both of which are implicated in the development of DR.

### Vascular Adhesion Protein-1 (VAP-1)

Vascular adhesion protein-1 (VAP-1) is a homodimeric sialylated glycoprotein expressed in vascular endothelial cells and involved in leukocyte transmigration (19–21). Previous studies have elucidated that VAP-1 is crucial in the pathology of systemic inflammatory diseases, including rheumatoid arthritis (22, 23), inflammatory bowel diseases (24), myocardial infarction (25), and diabetes (26). In ocular tissues, we reported that VAP-1 is localized to endothelial cells of retinal and choroidal vessels (27), and VAP-1 is involved in the molecular mechanisms of acute ocular inflammation and inflammation-associated ocular angiogenesis (28, 29). In addition, we also showed that VAP-1 blockade significantly reduced the transmigration and capillary entrapment of leukocytes in the retina in a diabetic animal model (30). Taken together, our prior research demonstrates that VAP-1 plays a role in the pathogenesis of DR by mediating leukocyte recruitment as a leukocyte adhesion molecule.

**Figure 2:**
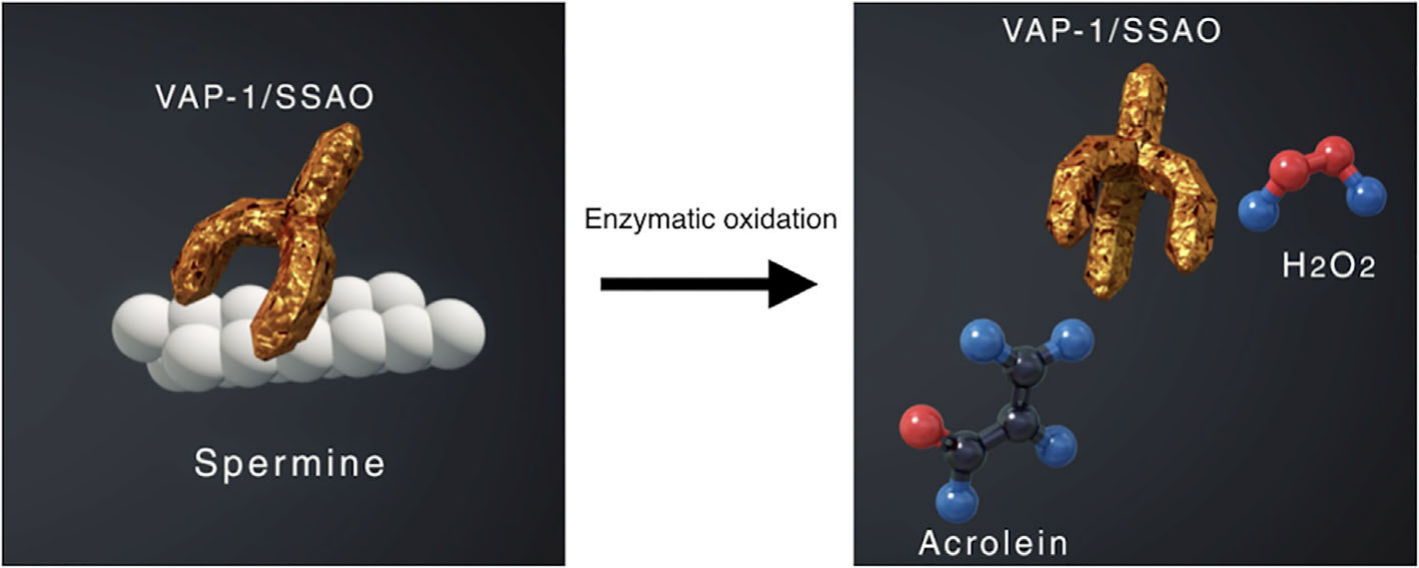
Enzymatic oxidation of spermine mediated by vascular adhesion protein-1 (VAP-1) function as semicarbazide-sensitive amine oxidase (SSAO). VAP-1 converts spermine to acrolein and hydrogen peroxide, both of which increase oxidative stress in the body.

**Figure 3:**
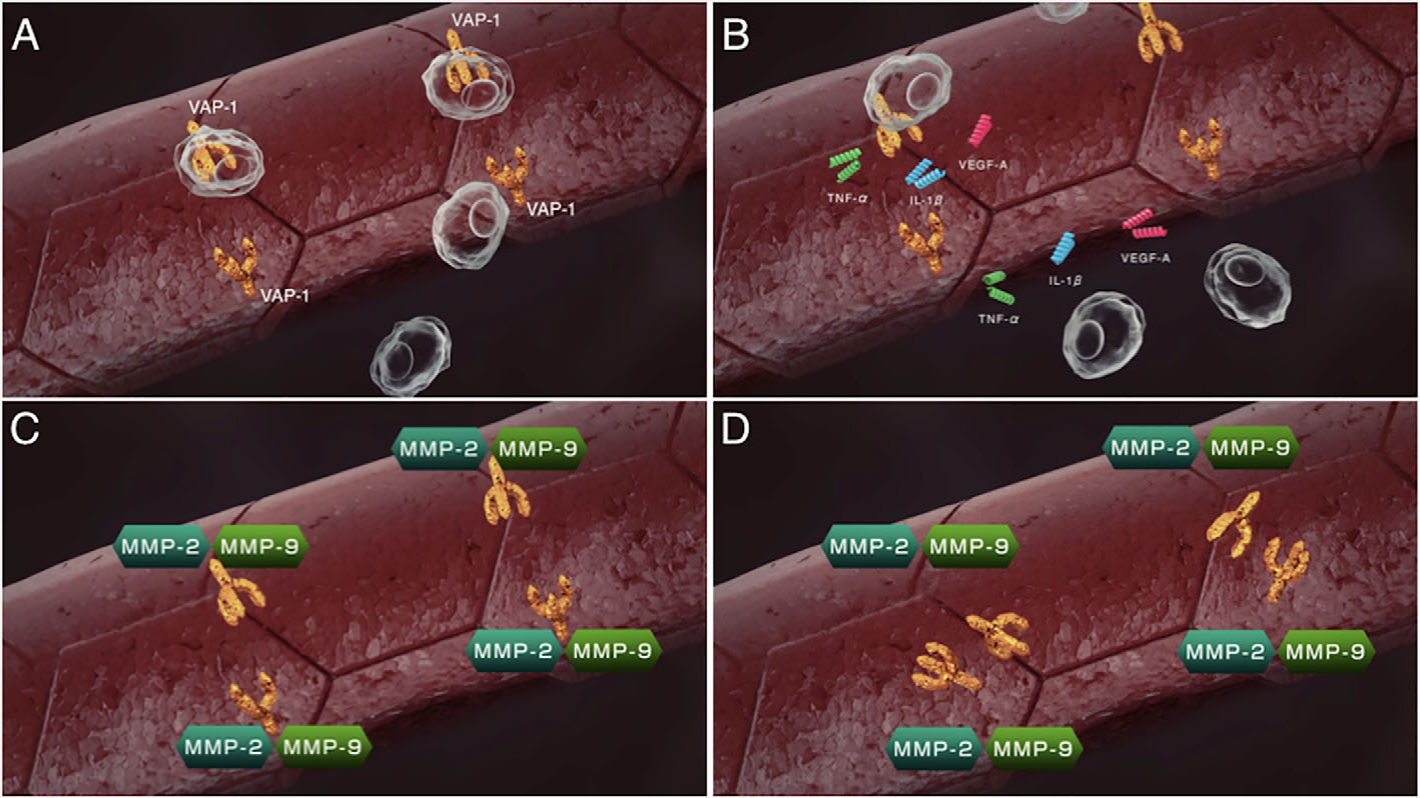
Sequential steps of release of the soluble form of VAP-1 from retinal capillary endothelial cells. **(A)** VAP-1 and other leukocyte adhesion molecules facilitate leukocyte recruitment upon inflammation. **(B)** Recruited leukocytes secrete inflammatory cytokines such as tumor necrosis factor-α (TNF-α), interleukin-1β(IL-1β) and vascular endothelial cell growth factor (VEGF). **(C)** MMP-2 and MMP-9 induced by the inflammatory cytokines proteolytically cleave the VAP-1 protein. **(D)** Soluble form of VAP-1 was released from the surface of endothelial cells.

VAP-1 also exists as a soluble form (sVAP-1) in mammals and is known to participate in theinitiation and development of systemic disorders (31–33). In addition to its role as anadhesion molecule, both membrane and soluble forms of VAP-1 function as semicarbazide-sensitiveamine oxidase (SSAO), which oxidizes aliphatic and aromatic primary monoamines and converts them tothe corresponding aldehydes with the release of hydrogen peroxide and ammonia (**Figure 2**) (34). Of note, oursubsequent analyses revealed that high glucose, inflammatory cytokines such as tumor necrosisfactor-α (TNF-α) and interleukin-1β (IL-1β) and angiogenic factor VEGF facilitate proteolytic cleavage of the membrane-bound VAP-1 from retinal capillary endothelial cells mediated by matrix metalloproteinase (MMP)-2 and MMP-9 (35, 36), both of which are important in fibrovascular tissue formation (**Figure 3**) (37). In addition, our *in vitro* study revealed that sVAP-1 mediates acrolein production *via* spermine metabolism, a polyamine in retinal endothelial cells (18). Polyamines are low molecular weight polycations that have two or more primary amine groups, and are known to play an important role in cell proliferation and differentiation (38). In mammals, there are three naturally occurring polyamines: putrescine, spermidine, and spermine (39). In patients with PDR, spermine levels are elevated 15-fold in the vitreous fluid compared to non-DR patients (40), indicating that the vitreous cavity is a substrate-enriched environment for sVAP-1. Indeed, sVAP-1 and FDP-Lys are increased and are correlated in the vitreous fluid of patients with PDR (18). Evidence suggests that acrolein is generated by intravitreal sVAP-1 released from retinal capillary endothelial cells in the presence of participant molecules such as inflammatory cytokines and proteinases in eyes with DR.

### Spermine Oxidase

**Figure 4:**
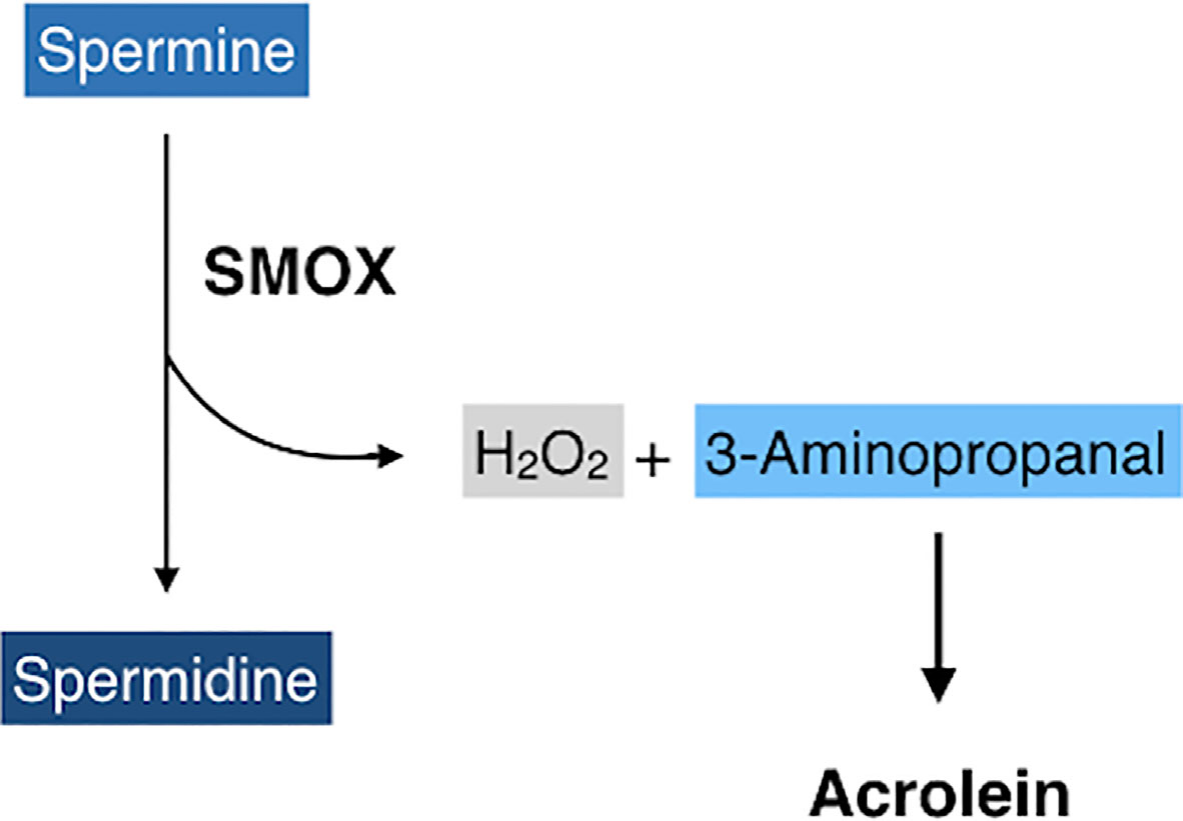
A schematic of oxidative degradation of spermine by spermine oxidase (SMOX). SMOX catalyzes spermine and produces spermidine, hydrogen peroxides, and 3-aminopropanal, which is non-enzymatically converted to acrolein.

Spermine oxidase (SMOX) is a flavin adenine dinucleotide-containing enzyme that catalyzes the oxidative degradation of spermine to produce spermidine, hydrogen peroxides, and 3-aminopropanal (41) that is non-enzymatically converted to acrolein (**Figure 4**) (42). SMOX is a highly inducible enzyme and its expression is upregulated by inflammatory cytokines, including TNF-α and IL-6 (43, 44), both of which are increased in eyes with PDR. Recent studies have revealed that FDP-Lys is accumulated in Müller glial cells of streptozotocin (STZ)-induced diabetic rats (45) and in migrated glial cells in the fibrovascular tissues obtained from patients with PDR (46). Therefore, both observation in humans and experimental evidence indicate that acrolein is generated and/or accumulates in retinal glial cells under diabetic conditions. However, retinal glial cells lack VAP-1 expression, and the exact mechanism of acrolein generation in these cells was unclear. Recently, our group discovered that SMOX mediates acrolein generation in cultured retinal glial cells, and that hypoxia induces SMOX production *via* HIF-1 binding to *SMOX* promoter (47). In addition, the localization of SMOX was seen predominantly in glial cells of fibrovascular tissues (47). In the advanced stage of DR, obliteration of retinal microvasculature elicits a decrease in tissue oxygen concentration (48, 49) and tissue hypoxia induces extensive cellular responses, including neovascularization that eventually results in proliferative changes at the vitreoretinal surface in eyes with DR. Our *in vitro* study revealed that hypoxia increases FDP-Lys and hydrogen peroxide levels in cultured retinal glial cells, which were abrogated by the potent SMOX inhibitor MDL72527 (47). Overall, the experimental evidence indicates that SMOX produces acrolein through the enzymatic conversion of spermine to spermidine in retinal glial cells under hypoxic conditions and presumably exacerbates oxidative stress in eyes with DR.

## Roles of Acrolein in DR

So far, it has been demonstrated that acrolein plays a significant role in the pathogenesis of systemic disorders, such as neurodegenerative diseases (50), cardiovascular diseases (51), and diabetes (52). With respect to diabetes, acrolein was reportedly increased in the serum and urine of patients with diabetes (52, 53). In the eye, previous studies demonstrated that FDP-Lys markedly increased in retinal glial cells of experimental diabetic rodents (45, 54, 55). We also reported that FDP-Lys was elevated in the vitreous fluid of patients with PDR (18). In addition, we found that FDP-Lys largely accumulated in glial cells (46) and endothelial cells (18) of fibrovascular tissues obtained from patients with PDR. Therefore, these data suggest that acrolein participates in the development of DR.

In the following section, we summarize the role of acrolein in the pathogenesis of DR.

### Inflammation

Recent studies have shown that chronic, low-grade inflammation underlies much of the vascularcomplications of DR (56, 57). Previously, it was demonstrated that acrolein activated the NF-κBpathway and induced pro-inflammatory cytokines including cyclooxygenase-2 (58). In addition, it was shown that acrolein induced pulmonary inflammation anddeath of lung epithelial cells *via* induction of NF-κB signaling in a mouse *in vivo* study (59). Acrolein also induced *TNF-α*, *IL-6*, and *IL-8* mRNA expression through NF-κB activation in human umbilical vein endothelial cells (HUVECs) (60).

In the retina, it was reported that acrolein induced transforming growth factor beta-1(TGFβ1), TGFβ2, and VEGF production in retinal pigment epithelium inhyperglycemic conditions (61). In contrast, anacrolein-scavenging agent significantly suppressed C-C motif chemokine ligand 2(*Ccl2*), *IL-1β*, and intercellular adhesion molecule 1 (*Icam1*) mRNA expression in the retina of diabetic rats (55). Accumulating evidence clearly indicates the involvement of acrolein in the inflammatory aspect of DR; however, further analysis is needed for the association between acrolein and pro-inflammatory cytokine production in DR.

### Increase in Oxidative Stress

The role of oxidative stress in the pathogenesis of DR has been extensively investigated in experimental and clinical studies. The levels of various reactive oxygen species (ROS), including superoxide (62) and hydrogen peroxide (63), have been shown to be elevated in the retina of diabetic animals. Conversely, the antioxidant defense systems in eyes of patients with DR have been shown to be functionally damaged (64). We previously demonstrated that acrolein reduces the intracellular reserves of GSH, leading to consequent increase in oxidative stress and cell death in retinal microvascular endothelial cells and glial cells (18, 65). Furthermore, the subsequent analysis further revealed that spermine oxidation by VAP-1 or SMOX results in acrolein generation and exacerbates oxidative stress in the microenvironment by both limiting the anti-oxidant defense system and enhancing ROS generation (18, 47). As aforementioned, VAP-1 and FDP-Lys are increased and are correlated in the vitreous fluid of patients with PDR (18). VAP-1 and N epsilon-(hexanoyl)lysine (HEL), an oxidative stress marker, are also correlated in the vitreous fluid from PDR patients (35). These data indicate that increased acrolein generated by VAP-1 and SMOX participates in the pathogenesis of DR.

### Glial Cell Activation

Glial cell activation is the initial response during the early stages of DR (66, 67) and retinal glial cells are known to proliferate and migrate into the vitreoretinal interface, which eventually leads to fibrovascular tissue formation during PDR progression (68, 69). Acrolein stimulated cell migration of Müller glial cells through induction of C-X-C motif chemokine ligand 1 (CXCL1) protein (65), a member of the C-X-C family of chemokines that promotes neutrophil and tumor cell migration through binding to the receptor C-X-C motif chemokine receptor 2 (CXCR2) (69, 70). It has been previously shown that the vitreous level of CXCL1 was increased in eyes affected by PDR (71) and in the retinal tissue of diabetic mice (72). In addition, we reported that CXCL1 was localized in GFAP-positive cells of fibrovascular tissues (65). Since FDP-Lys largely accumulates in the glial cells of fibrovascular tissues (46), it suggests that CXCL1 stimulates retinal glial cells in an autocrine fashion through its receptor, CXCR2, in response to acrolein under diabetic conditions (65). Previously, it was reported that an acrolein-scavenging agent could suppress glial fibrillary acidic protein (GFAP) expression, a representative marker of glial cell activation, in Müller glial cells of diabetic rats (55). Therefore, these data indicate that acrolein is potentially one of the molecules triggering retinal glial cell activation in diabetic eyes.

### Neurodegeneration

The retinal tissue consists of vascular and neural components. Whereas microvascular changes are integral to the formation and devastating effects of DR, DR has been recently recognized as a neuro-vascular disease. There is a growing body of evidence to indicate that neural components are damaged in the early phase of DR, even in the absence of vascular complications such as retinal hemorrhage and microaneurysm formation (73, 74). Basic research findings obtained from postmortem human specimens (75) and diabetic animals showed that diabetes causes cellular disturbance in neural cells, particularly in retinal ganglion cells (RGC) (76).

As mentioned, SMOX produces acrolein through its enzymatic activity. Liu et al. reported elevated levels of FDP-Lys in the ganglion cell layer and inner nuclear layer of the retina in a diabetic animal model (77). It was shown that SMOX inhibitor MDL72527 reduced the upregulation of FDP-Lys, retinal tissue thinning and RGC loss (77), suggesting that acrolein is potentially involved in the neurodegeneration in diabetic eyes.

## Therapeutic Approaches Targeting Acrolein

As described thus far, acrolein plays a significant role in DR and may be an important target for the prevention and treatment of DR. 2-Mercaptoethanesulfonate (MESNA) is a potent thiol based scavenger of acrolein that has already been used clinically to prevent urothelial toxicity by inactivating metabolites from antineoplastic agents, such as ifosfamide or cyclophosphamide (78). It was also reported that acrolein-scavenging agent 2-hydrazino-4,6-dimethylpyrimidine (2-HDP) reduced Müller cell gliosis and retinal inflammatory marker expression in STZ-induced diabetic rats (55). Alternatively, SMOX inhibitor MDL72527 treatment improved electroretinogram response and reduced retinal neurodegeneration in STZ-induced diabetic mice (77, 79). Therefore, inhibitors of acrolein and SMOX are potential therapeutic drugs for the treatment of DR.

## Conclusions

Previous studies have shown that levels of inflammatory cytokines and oxidative stress markers are elevated in the specimens of patients with DR, indicating the roles of the inflammatory process and oxidative stress in retinal microvascular complications caused by diabetes.

**Figure 5:**
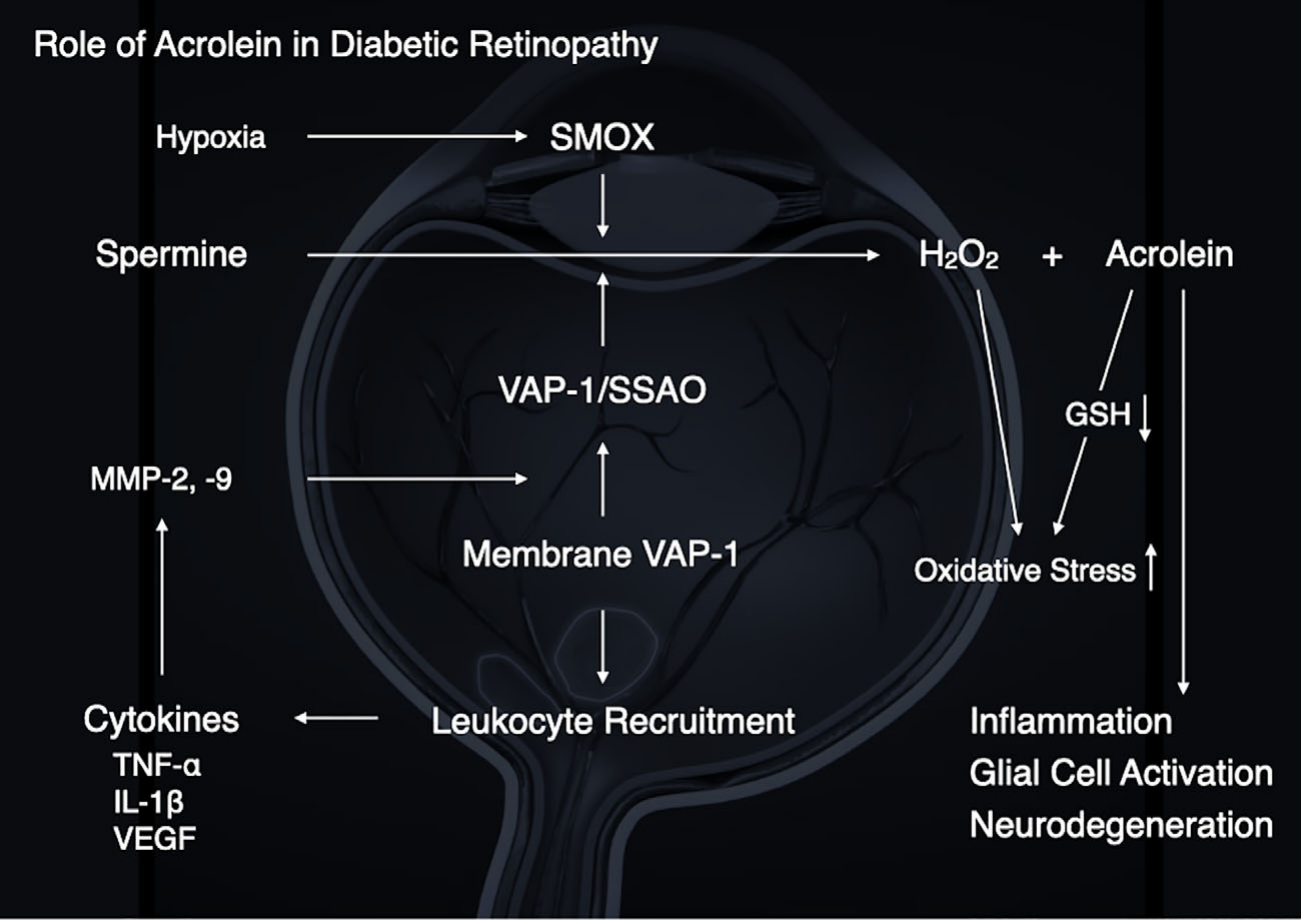
A schematic summary illustrating the role of acrolein in diabetic retinopathy.

The pathogenesis of DR is not entirely known. However, based on the preceding discussion, growing evidence suggests a pathological role for acrolein in the development of DR (**Figure 5**). Our group and others have demonstrated that diabetic conditions, which include high glucose, inflammation and hypoxia, enhance acrolein production through VAP-1/SSAO and SMOX induction, suggesting that acrolein contributes to the exacerbation of DR *via* enhancement of inflammation, oxidative stress, glial activation, and neurodegeneration.

## Author Contributions

MM wrote the paper. KN wrote and revised the paper. SI revised the paper. All authors contributed to the article and approved the submitted version.

## Funding

This work was supported by Grant-in-Aid for Scientific Research (B) [20H03837(SI)] and Grant-in-Aid for Scientific Research (C) [18K09393(KN) and 17K11442(MM)] of the Japan Society for the Promotion of Science.

## Conflict of Interest

The authors declare that the research was conducted in the absence of any commercial or financial relationships that could be construed as a potential conflict of interest.
